# A novel reverse transduction adenoviral array for the functional analysis of shRNA libraries

**DOI:** 10.1186/1471-2164-9-441

**Published:** 2008-09-24

**Authors:** Angelika Oehmig, Andrea Klotzbücher, Maria Thomas, Frank Weise, Ursula Hagner, Ralf Brundiers, Dirk Waldherr, Andreas Lingnau, Achim Knappik, Michael HG Kubbutat, Thomas O Joos, Hansjürgen Volkmer

**Affiliations:** 1 NMI Natural and Medical Sciences Institute at the University of Tübingen, Markwiesenstr. 55, 72770 Reutlingen, Germany; 2 ProQinase GmbH, Breisacher Str. 117, 79106 Freiburg, Germany; 3 MorphoSys AG/AbD Serotec, Lena-Christ-Str. 48, 82152 Martinsried, Germany

## Abstract

**Background:**

The identification of novel drug targets by assessing gene functions is most conveniently achieved by high-throughput loss-of-function RNA interference screening. There is a growing need to employ primary cells in such screenings, since they reflect the physiological situation more closely than transformed cell lines do. Highly miniaturized and parallelized approaches as exemplified by reverse transfection or transduction arrays meet these requirements, hence we verified the applicability of an adenoviral microarray for the elucidation of gene functions in primary cells.

**Results:**

Here, we present microarrays of infectious adenoviruses encoding short hairpin RNA (shRNA) as a new tool for gene function analysis. As an example to demonstrate its application, we chose shRNAs directed against seven selected human protein kinases, and we have performed quantitative analysis of phenotypical responses in primary human umbilical vein cells (HUVEC). These microarrays enabled us to infect the target cells in a parallelized and miniaturized procedure without significant cross-contamination: Viruses were reversibly immobilized in spots in such a way that the seeded cells were confined to the area of the viral spots, thus simplifying the subsequent addressing of genetically modified cells for analysis. Computer-assisted image analysis of fluorescence images was applied to analyze the cellular response after shRNA expression. Both the expression level of knock-down target proteins as well as the functional output as measured by caspase 3 activity and DNA fractionation (TUNEL) were quantified.

**Conclusion:**

We have developed an adenoviral microarray technique suitable for miniaturized and parallelized analysis of gene function. The practicability of this technique was demonstrated by the analysis of several kinases involved in the activation of programmed cell death, both in tumor cells and in primary cells.

## Background

In the years following the completion of the human genome sequencing projects, a variety of genome-wide screening procedures for functional analysis were developed. For instance, plasmid vectors were applied to transfer cDNA for over-expression, or, alternatively, shRNA knock-down cassettes for gene silencing [1]. To this end, different plasmid vectors complexed with transfection reagents were immobilized in microspots delivered to planar glass slides, which were subsequently covered with a monolayer of cells. The cells took up the genetic information (reverse transfection) and phenotypic alterations were functionally assayed [1]. However, many primary cells are not amenable to classical transfection protocols. In these cases viral vectors are the only means to genetically manipulate the target cells. This instigated us to test the suitability of viral vectors for arraying, for which purpose we have chosen adenovirus as a model. In the meantime, similar arraying experiments were carried out using lentivirus [2] and retrovirus [3].

We have generated a microarray of infectious adenovirus to convey genetic information into the target cells (reverse infection). By choosing an adequate blocking procedure, we managed to strictly confine the cells to the viral spots, preventing adherence to areas without genetic information. By co-expressing fluorescent markers, we could differentiate infected cells from non-infected cells, thus facilitating phenotypic analysis.

We have employed the adenoviral array technology to analyze the contribution of seven protein kinases to cell signalling in HUVEC after viral transfer of validated shRNA expression cassettes. This group of enzymes was selected to exemplify the application of miniaturized adenoviral arrays.

## Results

### Reverse transduction by arrayed adenovirus

**Figure 1:**
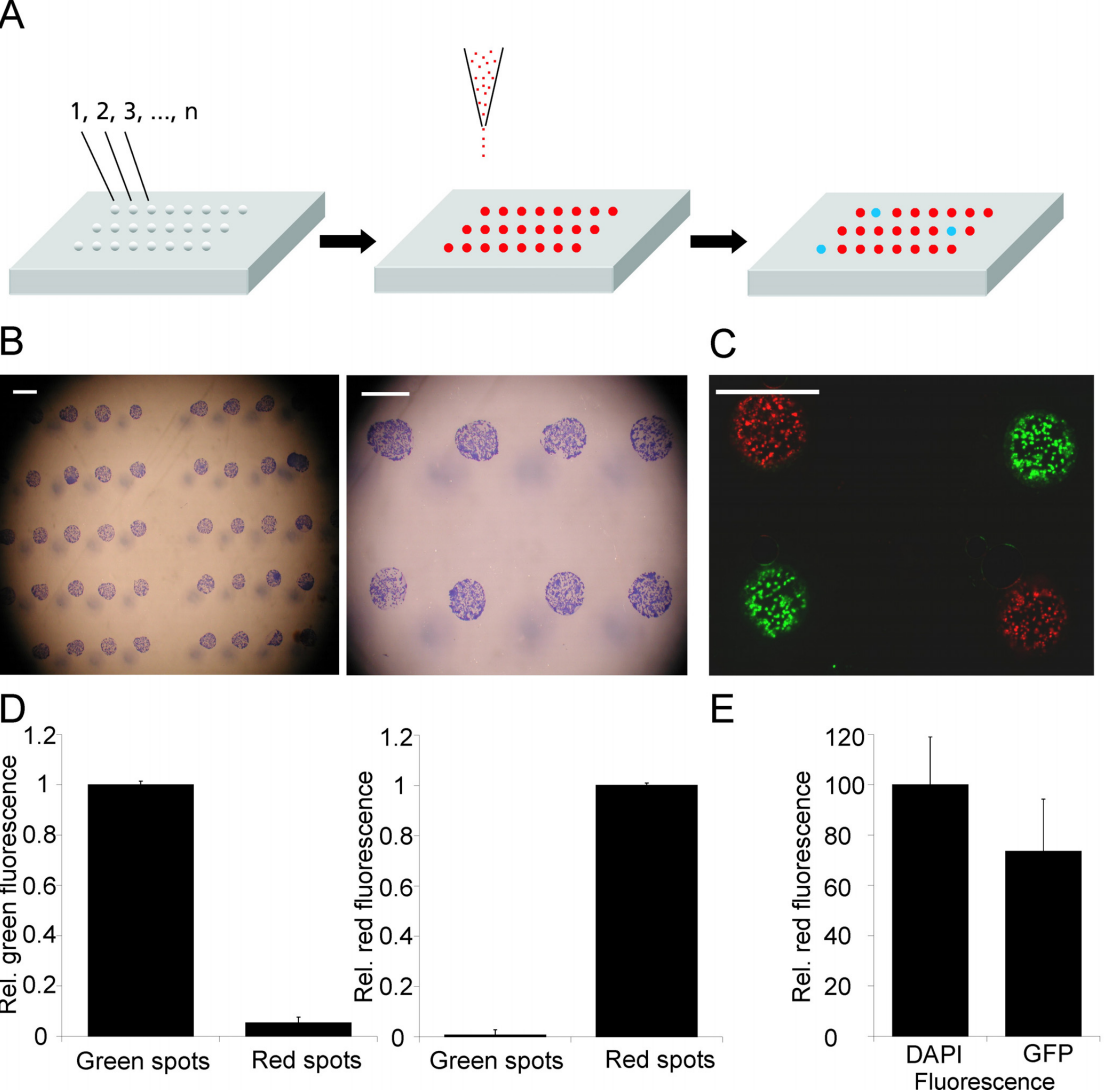
**Immobilization of viral spots in a microarray format**. **A**. Adenovirus encoding shRNAs directed against different target genes (1, 2, 3, ..., n) are immobilized on a glass slide. **B**. Coomassie staining of HeLa cells seeded on the adenoviral microarray. Due to the thickness of the carrier (glass-slide), shadows of the printed spots were cast on the stage plate of the microscope. This "shadowing" is not observed by using conventional fluorescence microscope, used for image analysis. Scale bar, 100 μm. **C**. Neighbouring spots of U-2 OS cells on the microarray. The cells were alternately infected with two types of adenoviruses, encoding either enhanced green fluorescent protein (EGFP) or red fluorescent protein (RFP), respectively. Scale bar, 100 μm. **D**. Quantitative analysis of viral spots, eight spots for each fluorescence marker, from Figure 1C for the detection of intermixture between neighbouring viral spots. **E**. Infection efficiency of U-2 OS cells: Percentage of the number of cells expressing EGFP compared to total number of cells as determined *via *DAPI stain (4 spots, n > 150).

The use of adenoviruses has been demonstrated for miniaturized high-throughput virus production as well as transduction in microwell plates [4]. A novel printing technique was developed to print arrayed adenovirus on glass slides for the subsequent infection of cells seeded on top (Fig. 1A). The viruses were immobilized in defined spots (diameter approx. 80 μm), and the surface outside the spots was blocked in a way that only the area of the viral spots was permissive to eukaryotic cell adhesion. Hence, the pattern of immobilized adenovirus was reflected by the formation of a corresponding cell array (Fig. 1A). This approach facilitates phenotypic analysis, as the cells to be investigated are spatially confined (Fig. 1B). Adenoviral vectors applied in Figure 1 were constructed to express enhanced green fluorescent protein (EGFP) or red fluorescent protein (RFP) as markers for the identification of infected cells. In addition, they contain Gateway™ recombination sites for high-throughput introduction of shRNA expression cassettes after in vitro recombination. Successful infection was assayed by EGFP or RFP fluorescence analysis. The appearance of EGFP/RFP-positive cells indicated that the immobilized viruses maintained their infectivity, and no significant intermixture occurred between neighboring viral spots (Fig. 1C). This finding was further supported by quantification of EGFP fluorescence in spots with RFP-expressing adenoviruses, and *vice versa *(Fig. 1D). Likewise, release of adenoviruses from spots was not observed when the washing buffer was tested for infectious particles (data not shown). Hence, the viruses were sufficiently immobilized to prevent cross-contamination between the spots, yet immobilized cells were still able to take up viral particles for infection.

The infectivity rate of reverse transduction was measured by comparing the number of EGFP-positive cells with the number of DAPI-stained nuclei. In the case of the osteosarcoma cell line U-2 OS, infection of approximately 70% of all cells was achieved (Fig. 1E). We found the chips with immobilized adenoviruses to remain infective for more than two weeks when stored at 4°C, without significant loss in overall infectivity (see Additional file 1).

In conclusion, arrayed adenoviruses are able to transduce cells efficiently, and the adenoviral arrays generated can be readily stored for prolonged time-periods without loosing infectivity, meaning this technology to be applicable for the large-scale applications.

### shRNA-mediated knock-down on a chip

**Figure 2:**
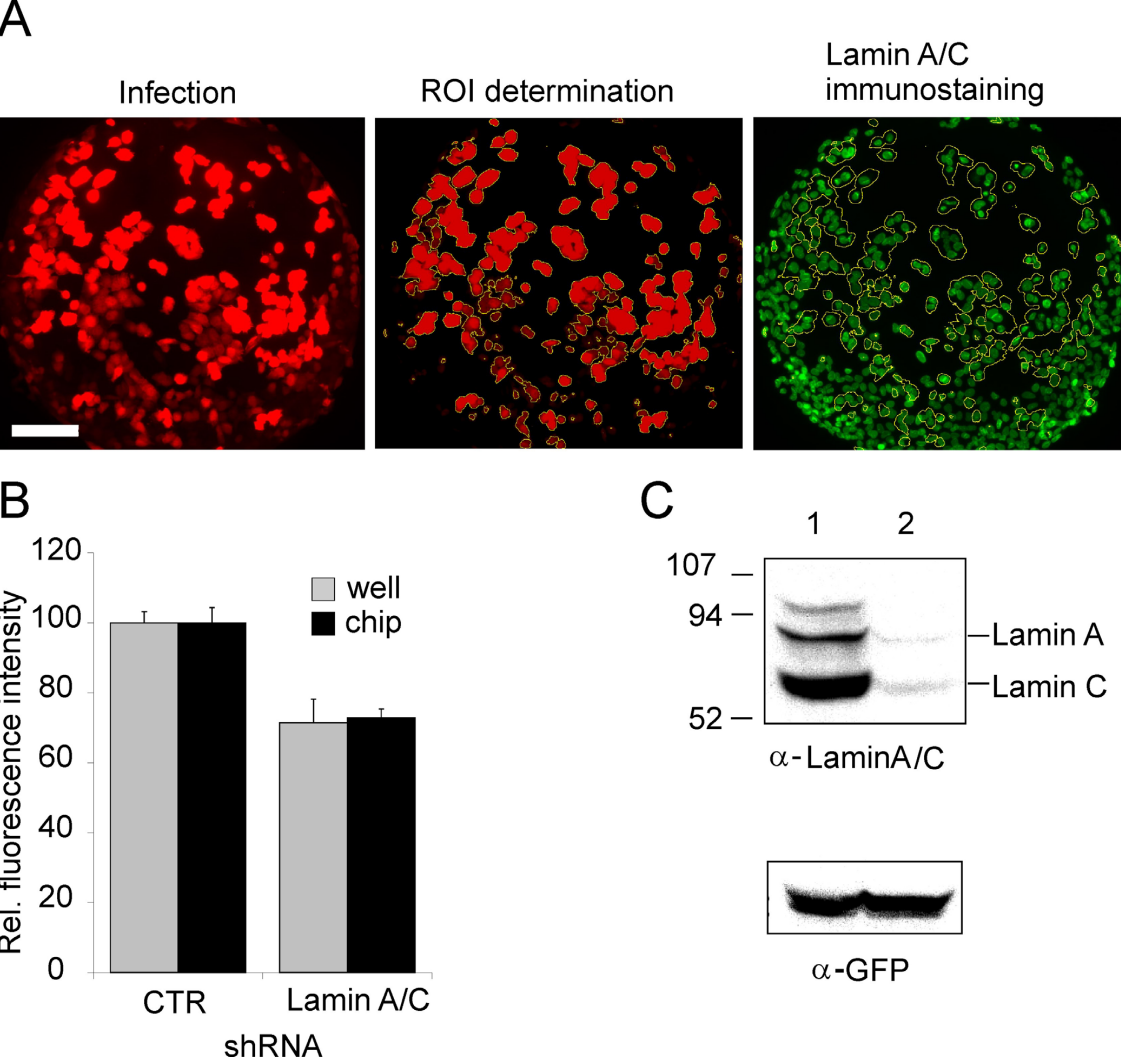
**Analysis of lamin knock-down in U-2 OS cells seeded on an adenoviral microarray**. **A**. Fluorescent micrographs of cells expressing RFP and shCTR stained with antibody directed against lamin A/C. Red fluorescence (left) indicates infected cells, green fluorescence (right) indicates lamin A/C expression. The diagram illustrates the evaluation procedure. Scale bar, 10 μm. **B**. Bar diagram, showing the averages of fluorescence intensity of the lamin A/C immunostaining of U-2 OS cells expressing either an irrelevant shRNA (shCTR), or shRNA directed against lamin A/C (shLamin) [5] for the experiments performed on the chip (black bars) or in the 96-well plate (gray bars). Infection efficiency achieved on the microarray as well as in the well suffices to elicit a significant knock-down (4 spots, n > 150, p < 0.005; by ANOVA analysis). **C**. Validation of shRNA targeting Lamin A/C by Western immunoblot analysis. U-2 OS cells were transfected either with pENTR +pcDNA3-eGFPneo (lane 1) or with pENTR-shLaminA/C +pcDNA3-eGFPneo (lane 2) and the lysates were immunoassayed 48 hours later.

An imaging procedure with the help of ImageJ  was used to calculate knock-down of target genes by quantitative analysis of immunstainings. The principles of the analysis are explained in Figure 2A. U-2 OS cells were seeded onto the adenoviral array, and infected cells were identified by RFP fluorescence (Fig. 2A, left image). Lamin A/C expression was visualized by indirect Cy2 immunofluorescence microscopy (Fig 2A, right image). For quantitative analysis, the fluorescence intensity of infected cells was determined by computer-assisted image analysis. In order to electronically evaluate the cells, the ROI (regions of interest) were defined using RFP fluorescence, indicative of adenoviral infection: To this end, the images were subjected to image segmentation. During the segmentation process, images were binarized by setting a defined threshold value for pixel intensity, and subsequently assigning the value of "black" to all pixels with an intensity above the threshold, and a value of "white" to all pixels with an intensity below the threshold. Hence, "black" pixels correspond to areas where infected cells are located, allowing to discriminate image regions containing infected cells from regions without infected cells, and the former regions gave rise to the "regions of interest" analysed further. Those ROIs (Fig 2A, center image) were superimposed on the corresponding Cy2 images, and the intensity of lamin A/C immunostaining was determined for each ROI (Fig 2A, right image).

Adenoviral vectors were arrayed on a chip encoding shRNA directed against lamin A/C (shLamin) [5] in addition to an RFP expression cassette. As a control, adenovirus encoding an irrelevant shRNA (shCTR) was introduced. This shCTR contains an shRNA sequence with no homology to human genes, as confirmed by BLAST search. Fluorescence intensities of the cells infected by the control adenovirus were compared to the one of the cells infected by the shLamin expressing virus as depicted in a bar chart (Fig. 2B). Compared to the control, lamin A/C expression was decreased in the case of the cells transduced with an adenoviral vector expressing lamin-specific shRNA, indicative of an overall reduction in lamin A/C expression.

Hence, our results show that shRNA-mediated knock-down is feasible in cells exposed to adenoviral arrays.

### Validating the adenoviral arrays in functional assays – comparison to conventional infection

**Figure 3:**
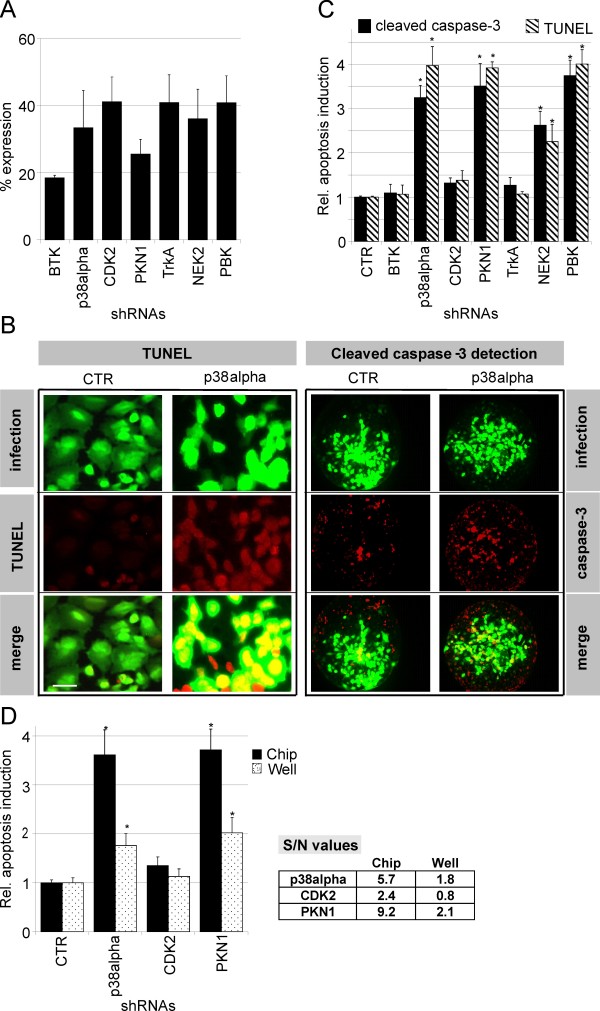
**Detection of apoptosis induction in U-2 OS cells seeded on an adenoviral microarray**. **A**. Relative expression levels of the target genes indicated after co-transfection of the psiCHECK marker plasmid and shRNA-encoding plasmids directed against the target genes indicated. **B**. TUNEL assay (left part of the image) or immunofluorescence of anti-cleaved caspase 3 (right part of the figure) of cells expressing EGFP and either control shRNA (left) or shRNA directed against p38alpha (right). Top, green fluorescence indicating infected cells; centre, red fluorescence for detection of TUNEL-positive cells or cells positive for cleaved caspase 3; bottom, merged images. Scale bar, 20 μm (for TUNEL) and 10 μm (for cleaved caspase 3 immunostaining). **C**. Functional assays performed on the microarray, using shRNA sequences directed against the target genes indicated. Caspase 3 (black bars) or TUNEL signal (hatched bars) were quantified in U-2 OS cells (ImageJ software). The statistical significance is marked by asterisks (4 spots, n > 150, p < 0.005, three independent experiments; by ANOVA analysis). **D**. Comparison of caspase 3 activation using either immunodetection on the microarray (black bars), or detection of enzymatic caspase 3 activity in cell lysates (Caspase-3-Glo, in a 96-well format (hatched bars). Statistical significance is marked by asterisks (4 spots, n > 150, p < 0.005, three independent experiments; by ANOVA analysis). On the right, the calculations of signal-to-noise ratios are shown.

A panel of seven adenoviral vectors expressing shRNAs directed against human protein kinases was applied to analyze the phenotypic response of tumor cells after gene silencing. The protein kinases selected are known to be involved in the regulation of apoptosis (BTK (Bruton's tyrosine kinase) [6-8], p38alpha (also known as MAPK14) [9-11]), in cell cycle progression (cyclin-dependent kinase 2, CDK2 [12-14], PKN1 [15]) or in mitotic regulation (NEK2 (also known as NIMA) [16-18] and PBK (PDZ binding kinase) [19,20]). Furthermore, one protein kinase was included for which no effect on apoptosis had been described (TrkA [21]). The shRNA sequences were validated and reduced protein kinase mRNA levels to ≤ 40% compared to the control shRNA (shCTR) in a reporter psiCheck™-2 assay (Fig. 3A). Luciferase activity was normalized to cells co-transfected with psiCHECK and shCTR. Three independent experiments have been carried out and the standard deviations are given. These kinase-specific shRNA adenoviral vectors were printed in arrays, and U-2 OS cells were immobilized as described above.

The induction of apoptosis was determined either by terminal-deoxynucleotidyl-transferase-mediated dUTP-biotin nick end labelling (TUNEL), or by immunodetection of cleaved caspase 3. A representative set of micrographs is given in Figure 3B, in which infected cells were identified using EGFP fluorescence (Fig. 3B, top images), whereas red fluorescence indicated incorporation of rhodamine-labelled dUTP or the presence of activated caspase-3 (TUNEL or cleaved caspase-3 staining, Fig. 3B, centre images). A merger of both micrographs is likewise given (Fig. 3B, lower images), in which yellow indicates a strong TUNEL- or caspase 3-signal. As a result, a strong induction of cell death upon knock-down of p38alpha in U-2 OS cells was detected.

Fluorescence intensities of both activated (cleaved) caspase 3 and TUNEL in comparison to cells infected with the control adenovirus (shCTR) were quantified as outlined above. The induction factor observed using activated caspase 3 or TUNEL staining as a determinant was up to 4-fold (Fig 3C). The knock-down of the four kinases p38alpha, PKN1, NEK2 and PBK evoked apoptosis as determined via caspase 3 activation compared to the control infection (p < 0.005). The same four kinases were identified as targets using the TUNEL assay (p < 0.005). Hence, using two independent assays for apoptosis detection, we could demonstrate the feasibility of functional assays on the adenoviral chips.

In Figure 3D, the functional assay performed on the adenoviral array was compared with the conventional assay using transfection of U-2 OS cells in a 96 well format: The induction of apoptosis on the array was determined by the detection of activated caspase 3 as described above, whereas the induction of apoptosis in the 96-well plate was determined by measuring the enzymatic activity of caspase 3 using a proluminescent caspase 3 substrate (Caspase-Glo^® ^3/7 Assay, Promega), which is adapted to high-throughput cellular assay systems. In this kind of assay, the generated luminescence is proportional to the amount of caspase activity present. For comparison, shRNA sequences against two anti-apoptotic kinases, p38alpha and PKN1, as well as an shRNA sequence against CDK2, the knock-down of which did not induce apoptosis, were chosen, and the induction of caspase 3 activity was depicted. Induction of apoptosis after shRNA-mediated knock-down of p38alpha and PKN1 exceeded the signals of the well-based assay which indicated that the adenoviral chip is a suitable alternative to the well-based assays. No induction of apoptosis was observed with either assay after expression of the vector specific for the knock-down of CDK2.

The signal-to-noise ratios (S/N) in Fig. 3D were calculated as a ratio of the difference between mean signal and mean background values to the background standard deviation [22] in order to compare the sensitivity of the on-chip assay with the in-well assays. For the experiment performed on the chip, they amount to: 5.7 (p38alpha); 2.4 (CDK2); 9.2 (PKN1). For the in-well trials, they amount to: 1.2 (p38alpha); 0.8 (CDK2); 2.1 (PKN1). Similar experiments using TUNEL assay were not performed.

### Functional assays using primary HUVEC

**Figure 4:**
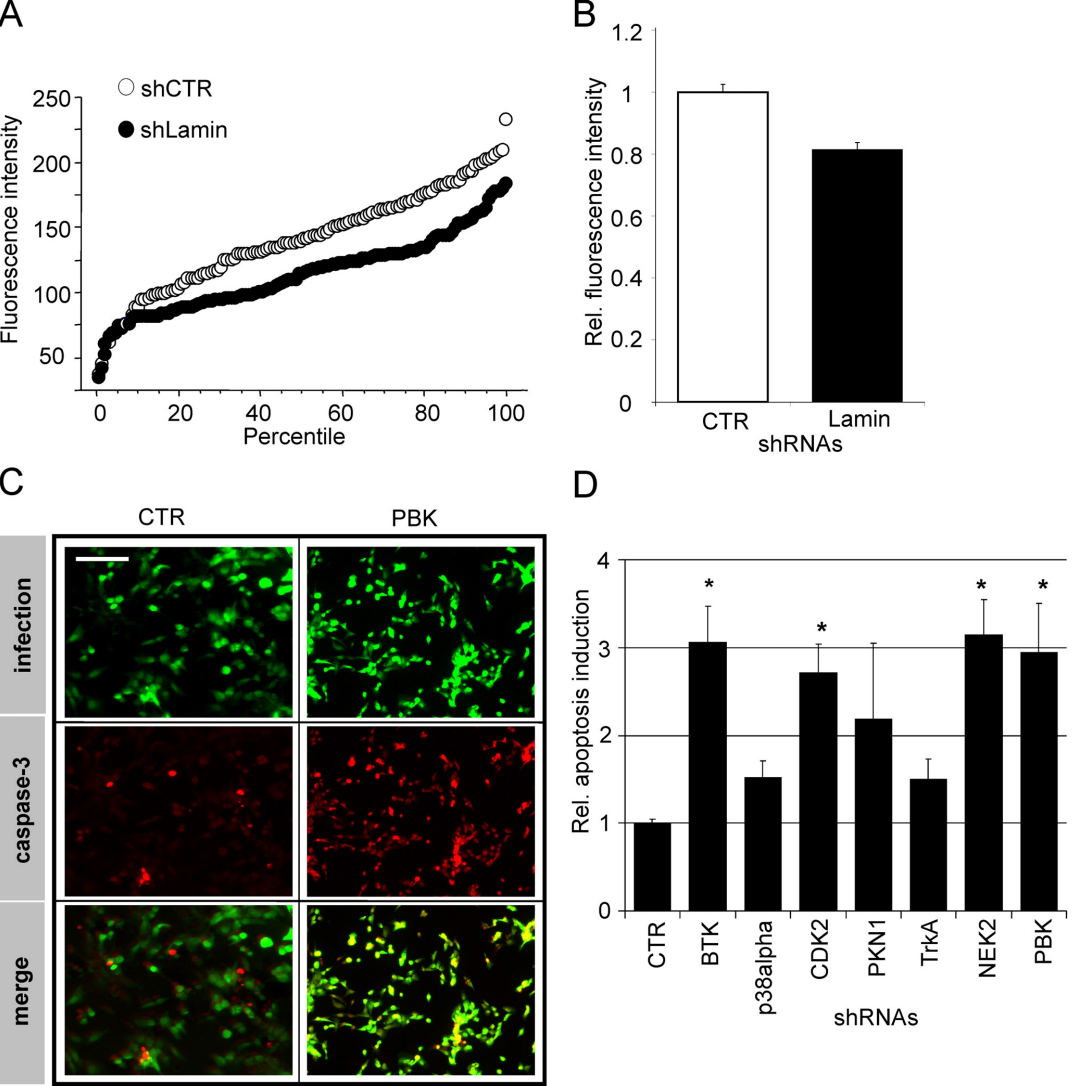
**Application of the adenoviral microarray to primary human cells (HUVEC)**. **A**. Percentile diagram showing the distribution of immunofluorescence intensity of HUVEC, indicative of lamin A/C: Cells were infected with adenovirus encoding either shCTR (open circles) or shLamin (closed circles) (p < 0.005; n > 150; by ANOVA analysis). **B**. Bar chart corresponding to the Lamin A/C expression levels shown in Fig. 4A: shCTR (white bars), shLamin (black bars) (p < 0.005; n > 150; by ANOVA analysis). **C**. Representative immunofluorescent images of HUVEC cells analysed in Figure 4D: Cells were infected with adenovirus encoding either shCTR (left), or shPBK (right). Green fluorescence (top) indicates infected cells, red fluorescence (middle) is indicative of activated caspase 3, merged images (bottom). Scale bar, 20 μm. **D**. Detection of apoptosis by immunostaining of α-cleaved caspase 3, using adenoviruses encoding specific shRNAs as indicated. The asterisks mark significant induction of apoptosis (4 spots, n > 150, p < 0.005; three independent experiments; by ANOVA analysis). TUNEL assay was not performed using HUVEC cells.

The advantage of adenoviral infection lies in the fact that adenoviruses allow the genetic manipulation of cells not amenable to classical transfection protocols, such as many primary cells. To make use of this advantage, we investigated both the efficiency of the knock-down and the employability of a functional assay using primary human umbilical vein endothelial cells (HUVEC). The knock-down efficiency was analyzed by seeding HUVEC onto a microspot array of immobilized adenovirus encoding RFP as well as shRNA directed against lamin A/C (shLamin). As a control, adenoviruses encoding RFP as well as a control shRNA (shCTR) were likewise printed. A comparative quantitative analysis was conducted as described above, the result of which is presented in a percentile diagram (Fig. 4A) as well as in a bar chart (Fig. 4B). Compared to the control situation, a significant reduction of fluorescence intensities was observed in cells infected with adenovirus expressing shRNA specific for lamin A/C. Hence, the overall reduction in lamin A/C expression indicated that adenoviral arrays are also applicable to human primary cells. Adenoviral arrays yielded levels of lamin A/C knock-down of 30–40% in HUVEC cells, which were quantitatively similar to the levels observed in U-2 OS cells.

Next, we asked whether an array of kinase-specific shRNA expressing adenoviruses would result in detectable induction of apoptosis in HUVEC. As shown above for U-2 OS cells, HUVEC were exposed to arrayed adenoviral shRNA vectors and activated caspase 3 was detected by immunofluorescence analysis. Figure 4C demonstrates induction of apoptosis in HUVEC after infection with shRNA directed against PBK. Here, similar to Figure 3B, infected cells were identified using EGFP fluorescence (Fig. 4C, top images), whereas red fluorescence indicated incorporation of Cy3-labelled antibodies against cleaved caspase 3 (Fig. 4C, centre images). A merger of both micrographs is likewise given (Fig. 4C, lower images), in which yellow indicates a strong activation of programmed cell death. As can be inferred from Figure 4D, the maximal induction of apoptosis was approx. 3-fold in comparison to the control vector (shCTR). The induction factors observed were significantly different when compared to the control situation (p < 0.005) and within a similar range as those found after transduction of U-2 OS cells.

Whereas the knock-down of NEK2 and PBK induced apoptosis in both HUVEC and U-2 OS cells, the cells differed in their response to the knock-down viruses against BTK, CDK2, p38alpha1, and PKN1: The knock-down of the former resulted in apoptosis in HUVEC, and the knock-down of the latter evoked apoptosis in U-2 OS.

In conclusion, we show that adenoviral arrays are suitable to perform functional assays even in primary cells. Hence, the adenoviral microarray allows a comparative analysis of kinase functions in different cell types under miniaturized and parallelized conditions.

## Discussion

Multiple RNAi platforms are now available for performing large-scale loss of function screens on mammalian cells. Viral-based screens have been introduced for their promise in expanding RNAi screens to models incompatible with conventional siRNA-based approaches.

We present here a microarray suitable for miniaturized and parallelized genetic manipulation and screening of primary human cells. As a vector for gene transfer we have employed adenoviruses. The viral vector system applied needs to include stable viral particles to enable long-term storage and transport. Additionally, high-throughput applications for genome-wide screenings require the possibility to produce virus in a small scale, miniaturized format, properties which are adequately met by adenoviral vectors.

In addition to the adenoviral microarray presented here, a similar system was also reported for lentiviral [2] and retroviral [3] shRNA expression vectors. Adenoviral and lentiviral screening platforms may be advantageous when assays are to be performed in primary, nondividing/differentiated cells or in established cell lines refractory to transfection. The technology of viral immobilization is comparable in all the cases: they rely on the principle of "reverse transduction/infection". The viral stocks are printed manually or automatically on glass slides coated with γ-amino propyl silane in the case of lentiviral microarrays, with a nanostructured titanium dioxide film in the case of retroviral microarrays, or with nitrocellulose for adenoviral microarrays. These printed chips can be stored or used directly. The production of high-titer viral particles in miniaturized 96-well format had earlier been demonstrated for lentiviruses [23,24], retroviruses [25] and for adenoviruses [26].

The decision as to which technology of viral delivery to employ will be situational as each of these three approaches comes along with distinct advantages and drawbacks. The advantage of the adenoviral microarray system presented in this study is an application of an appropriate blocking solution after printing the adenovirus. This implementation serves to confine cell adherence to the distinct spots containing the genetic information. Such confinement resulted in an array of cells, significantly simplifying identification in subsequent analysis. Additionally, it results in a reduced number of cells required for screening, which is especially relevant for the screening of primary cells, as the amount of cells available is frequently restricted. Whether the blocking step is applicable to the lentiviral microarray or any other viral system remains to be shown. As the viral vector system applied is characterised by highly stable viral particles, storage and transport of ready-to-use arrays is also feasible.

Genetic manipulations carried out in a microwell format allow for the well-by-well analysis using biochemical assays, the sensitivity of which needed to be achieved in a microarray configuration by fluorescence imaging. We have hence compared a sensitive well-based assay measuring the induction of apoptosis via enzymatic activity of caspase 3 in the lysate with immunodetection of activated caspase 3 on the microarray. As judged by the signal-to-noise ratios, the microarray-based assay provided signals at least in the range of the in-well assay. For optimal sensitivity, it should be noted that any assay carried out on a microarray requires discrimination between cells which have taken up shRNA constructs and untransduced cells. Here we introduced adenoviral vectors which enable the required discriminatory power, provided by a suitable marker incorporated in the vector backbone.

In order to test the feasibility of the adenovirus array technology for unravelling signalling pathways in a model primary cell, we studied the influence of shRNA-mediated knock-down of a set of seven protein kinases on the apoptotic process. The applicability of the device for functional assays on primary cells was exemplified using HUVEC. The comparison of signals obtained infecting HUVEC or U-2 OS cells revealed cell-type-specific responses to the knock-down constructs directed against individual kinases. The technology of the adenoviral siRNA microarray allowed to quantitatively measure phenotypical differences between these two distinct cell lines and therefore was qualified as a convenient and suitable tool for the cell-based analysis. Nevertheless, for more fundamental conclusions of the biological impact of these kinases, a more detailed investigation is needed, e.g. by testing several independent shRNA sequences directed against the targets identified to rule out off-target effects.

The kinases analyzed have been chosen from two categories: On one hand, we have selected rather general interplayers, such as PBK and NEK2, which have been shown to be important for cell cycle progression, DNA damage, and mitotic regulation [16,17,19,20]. On the other hand, we have selected more specific interplayers: p38alpha, BTK, PKN1 and CDK2 [6,8,9,12,13,15,27-29]. The knock-down constructs against PBK and NEK2 drove both HUVEC and U-2 OS cells into apoptosis, consistent with their overall importance for cell cycle progression.

A more specific response was observed for p38alpha and PKN1 in U-2 OS cells. These experiments suggest that osteosarcoma cells exhibit a higher sensitivity towards inhibition of kinases involved in the MAPK pathway than HUVEC. On the contrary, the knock-down of the kinases BTK and CDK2 elicited apoptosis in HUVEC but not in U-2 OS cells. As it was reported by Cai et al., solely depleting CDK2 was not sufficient to induce apoptosis in the osteosarcoma cell line U-2 OS [12].

The depletion of TrkA (also known as Ntrk1; neurotrophic tyrosine kinase, receptor, type 1) showed no effect on the induction of apoptosis in either cell type. This kinase depends on a particular surface membrane receptor for signal transmission, which might be absent or inactive in the cells examined [21].

## Conclusion

In general, the results on apoptosis induction upon knock-down of particular targets using the adenoviral chip correlated with published data [8,13,15,16,18,19,21,27,28,30]. Additionally, the functional assays performed using this practical microarray technique allowed the preliminary identification of several genes involved in the activation of apoptosis, both in tumor and in primary cells. The easy handling of the adenoviral microarrays, their suitability for immunochemical studies and for other diverse applications, as well as the possibility to acquire reliable results within a short period of time – these are the advantages of the present system. In addition, the suitability of adenoviral microarrays for miniaturized and parallelized screening makes them an attractive tool for a variety of scientific purposes.

## Methods

### Cell lines

HEK293 cells (CRL-1573, ATCC) and U-2 OS cells (HTB-96, ATCC) were cultivated in DMEM (Gibco), complemented with 200 mM Glutamin and 10% FCS (HyClone). HUVEC (PromoCell) were cultivated in EBM-2 Bulletkit medium (Cambrex).

### Design and cloning of shRNA sequences

The shRNA sequences directed against human kinases were based on siRNA sequences selected using public servers such as the Whitehead siRNA selection program [31], the Sfold siRNA design methodology program [32], the GenScript siRNA Target Finder , and the Dharmacon siDESIGN^® ^Center . The shRNA sequences are available upon request.

For the generation of shRNA-expressing vectors, oligonucleotide pairs were synthesized encompassing the siRNA sequences (19 nt or 21 nt) and their reversely complementary sequences, connected by the spacer 5'-CGAA-3'. Immediately downstream of the complementary sequences, there is a stretch of five thymidine residues, which serves as a stop signal for RNA polymerase III. Hence, a transcript is generated that can form a hairpin structure, in which the complementary sequences form the stem and the spacer forms the loop [33,34].

**Figure 5:**
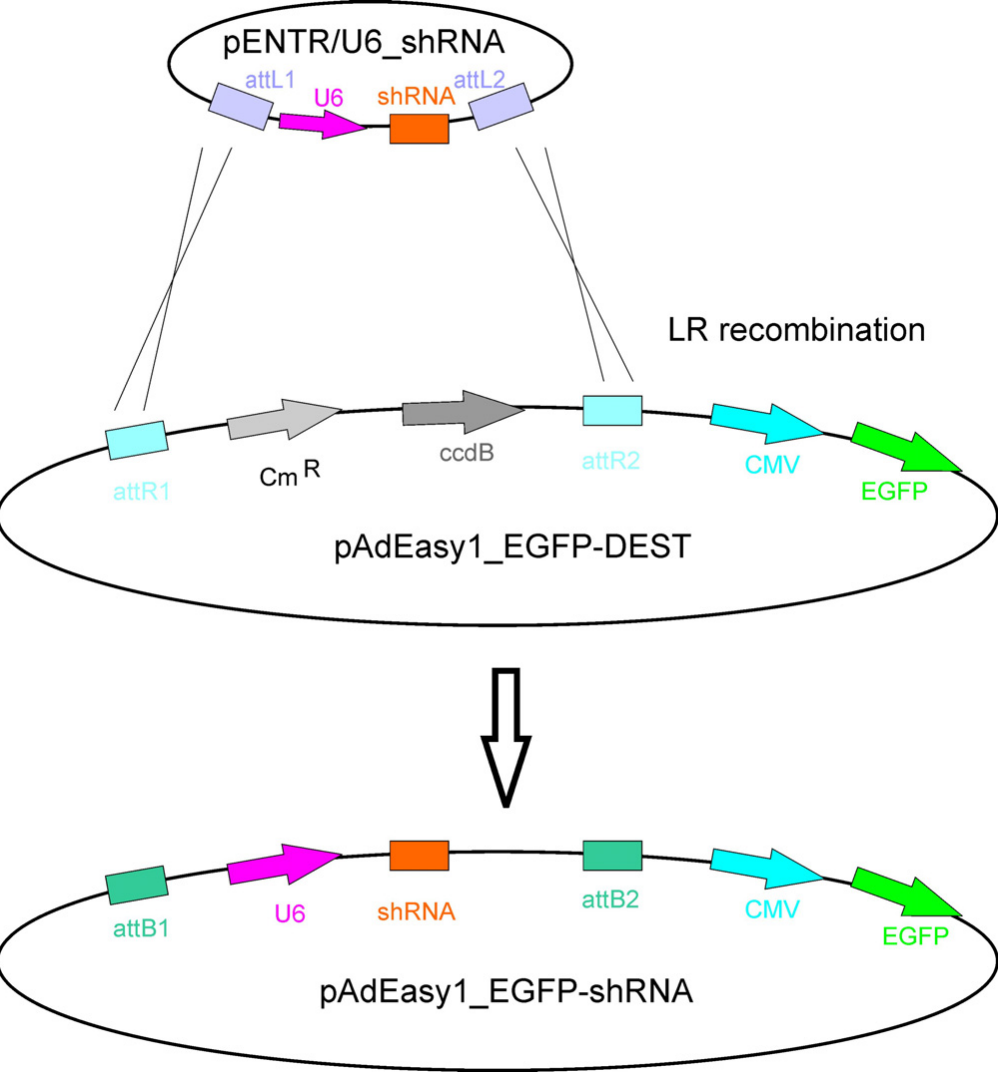
**Transfer of validated shRNA expression cassettes into the adenoviral genome**. Site-specific recombination between attL1/2 and attR1/2 from the entry vector pENTR/U6_shRNA into the adenoviral genome pAdEasy-1_EGFP-DEST.

The oligonucleotide pairs were annealed and ligated into pENTR™/U6 according to the manufacturer's protocol (BLOCK-iT™ U6 RNAi Entry Vector Kit, K4945-00, Invitrogen). In these vectors, the expression cassette – consisting of the human U6 promoter and the shRNA sequences – is flanked by the attachment sites attL1 and attL2, which allow the transfer of the expression cassette into the adenoviral destination vectors by site-specific LR recombination. The vector pAdEasy1_EGFP_shRNA is used to raise adenoviral stocks for printing as described below. A similar construct encoding RFP was likewise generated (Fig. 5).

### Validation of shRNA sequences

For each kinase, the cDNA was cloned – sometimes only partially – between the open reading frame of the humanized *Renilla *luciferase gene and a synthetic polyadenylation site of plasmid psiCHECK™-2 (C8021, Promega, Genebank accession # AY535007). Depending on the presence of suitable restriction sites upstream and downstream of the kinase open reading frames, modified versions of psiCHECK™-2 were generated with extended multiple cloning sites. If no suitable restriction sites were present, the cDNAs were amplified by PCR using primers containing the desired restriction enzyme recognition sites.

For the validation of the efficiency of the knock-down, HEK293 cells were co-transfected with a psiCHECK™-2 derivative containing the target cDNA sequence and the corresponding pENTR™/U6 plasmid encoding the shRNA. The co-transfection was carried out in a 96-well format, with 0.2 μg DNA/well using Lipofectamine™ 2000 Transfection Reagent (11668-019, Invitrogen) according to the manufacturer's protocol. Approx. 48 hours after transfection, the cells were lysed and the luciferase activity was determined according to the manufacturer's instructions (Dual-Glo™ Luciferase Assay System, E2920, Promega). Only those shRNA sequences which reduced luciferase activity by > 50% were transferred into a destination vector to generate the corresponding adenoviral shRNA expression vector.

### Western Blotting

The U-2 OS cells were transfected with the indicated constructs and 48 hours later the cells were lysed using Laemmli Buffer, containing 2% SDS, 10% glycerol, 5% 2-mercapto ethanol, 0,002% bromphenol blue and 0,075 M Tris HCl. Correlative to the transfection efficiency, an appropriate amounts of the protein lysate were loaded on 10% SDS-PAGE gel. After transfer and blocking procedures, the nitrocellulose membrane was incubated with mouse anti-Lamin A/C antibody (1:5000 diluted, Nr. 05714, Upstate Biotechnology) following immunostaining with HRP-conjugated rabbit anti-mouse secondary antibody. The final blots were treated using ECL™ Plus Western Blot Detection Reagent (RPN2132, GE Healthcare Life Sciences) for the detection of the signal.

### Construction of adenoviral shRNA expression vectors

The adenoviral destination vectors used were pAdEasy1_EGFP-DEST and pAdEasy1_RFP-DEST, which contains the *attR1*-Cm^R^-*ccdB*-*attR2 *cassette upstream of the cassette for the expression of the marker genes *enhanced green fluorescent protein *(EGFP) or *red fluorescent protein *(RFP), respectively. These adenoviral vectors are based on pAdEasy-1 (Stratagene), into which the above mentioned cassettes were cloned by homologous recombination using *Escherichia coli *BJ5183[35]. Furthermore, the original ampicillin resistance gene was replaced by a tetracycline resistance gene.

The *attR1*-Cm^R^-*ccdB*-*attR2 *cassette was generated by BP recombination between pDONR221 (Invitrogen) and a suitable cassette containing *attB1 *and *attB2*, using the Gateway^® ^BP Clonase™ II enzyme mix (11789020, Invitrogen) according to the manufacturer's instructions.

The EGFP expression cassette originated from pEGFP-N1 (Clontech; GenBank Accession # U55762) and contains the immediate early promoter of CMV, the EGFP open reading frame as well as the SV40 polyadenylation signal. The RFP expression cassette originated from pDsRed-Express-N1 (Clontech) and contains the immediate early promoter of CMV, the RFP open reading frame as well as the SV40 polyadenylation signal.

The shRNA expression cassettes in pENTR™/U6 were transferred into the adenoviral destination vectors by site specific recombination *in vitro *(Gateway^® ^LR Clonase™ II Enzyme Mix (11791020, Invitrogen).

The resulting vector pAdEasy1_EGFP_shRNA carries the shRNA expression cassette as well as a marker gene under the control of the CMV promoter. Hence, the cassette containing the genes for Chloramphenicol resistance (Cm^R^) and the bacterial *ccdB *gene is replaced by the shRNA expression cassette. Concomitantly, the Cm^R^-*ccdB *cassette is transferred to the pENTR vector (Fig. 5).

### Raising of adenoviral stocks and printing of adenoviral arrays

The adenoviral vector system used is based on the genome of the human adenovirus serotype 5 (Ad5), from which the early genes E1 and E3 have been deleted (AdEasy™ Adenoviral Vector System, Stratagene) [35,36]. Adenoviruses were raised from HEK293 cells according to the manufacturer's instructions in three rounds of amplification. Briefly, HEK293 cells were transfected with corresponding pAdEasy-CMV vector using Lipofecatmine 2000 (11668, Invitrogen) in a 6-well plate and the cells were scraped off 13 days later. After a short centrifugation by 2000 rpm, the cells were resuspended in 1 ml of PBS and subsequently submitted to 4 freeze-thaw cycles in liquid nitrogen. For the first amplification 50% of the cells were transferred to a 10 cm dish with HEK293 cells. The above described procedure was repeated 10 days later for the second amplification and 50% of the cells were used for the infection of three 10 cm dishes. Finally, three days later the procedure of virus concentration was repeated for the third amplification and 50% of cells were used to infect 40 10 cm dishes. 5–7 days later the same procedure of virus extraction was performed, the pellet was resuspended in 14 ml of PBS and an additional centrifugation by 10.000 rpm for 30 minutes was introduced after freeze-thaw cycles. The supernatant was transferred in to new tubes and frozen at -70°C.

The titres obtained after the third amplification were in the range of 1 × 10^9 ^infectious units/ml. The adenoviral stocks could be stored at -70°C over months without loosing their infectious capacity by -70 C. The procedure of virus generation is non-laborious and suitable for high-throughput applications.

Adenoviral stocks, resuspended in PBS, were printed onto glass slides covered with nitrocellulose, using a manual 8-pin arrayer (MicroCASTer™, Schleicher & Schuell). Nitrocellulose covering was achieved by dipping the microscopic slides in a 25 mg/ml solution of nitrocellulose (N8267, Sigma-Aldrich) in methanol and subsequent evaporation of the solvent under sterile conditions using a laminar air flow bench.

The printed adenoviral chips were shown to be not impaired in their infectious capacity till day 17 by storing at 4°C. Longer periods of storage are not recommended since the viruses gradually loose their infectious capacity.

### Infection on the chip and functional assays

After printing, the surface outside the viral spots was blocked by the application of 1 ml of StabilGuard^® ^(SG01, SurModics, Eden Prairie, MN, USA) [37,38], passed through a 0,2 μ filter, for 45 minutes and the slides were subsequently washed with phosphate-buffered saline (PBS).

4–5 × 10^5 ^trypsinized cells were distributed over the surface of the chip, which was placed into a Petri dish. The cells were left for 8–10 hours at 37°C in 5% CO_2_. Then, fresh medium was added to the cells so that the chip was completely covered with medium. Three (in the case of cleaved caspase 3 measurement) or four (in the case of the TUNEL assays) days later, the chips were washed twice with PBS and fixed in 4% formaldehyde solution for 15 minutes. After a subsequent wash with PBS, the cells on the chips were permeabilized using 0.25% Triton X-100 in PBS for 15 min. To block unspecific binding of the antibodies, the cells were blocked with 1% BSA (bovine serum albumin) in PBS/Tween-20 for 30 minutes. Afterwards, the chips were treated with either anti-lamin A/C monoclonal mouse antibody (SC-7292, Santa Cruz) or with anti-cleaved-caspase-3 (Asp175) antibody (9661, Cell Signalling Technologies), diluted in 1% BSA-PBS solution, for at least one hour at 37°C. After decanting the first antibody solution, the chips were washed three times with PBS and incubated for 1 hour with secondary Cy2-coupled goat anti-mouse antibody for lamin staining (115-226-003, Dianova), or with Cy3-coupled goat anti-mouse antibody for caspase-3 staining (115-116-003, Dianova). Prior to mounting with DacoCytomation fluorescent mounting medium (S3023, DakoCytomation), the chips were counterstained for 1 minute with DAPI followed by fluorescent microscopy analysis.

The TUNEL assay was performed using the In Situ Cell Death Detection Kit, TMR Red (Roche Applied Science, 2156792) exactly following the manufacturer's instructions. Shortly, 4 days after infection, the chips were fixed using the Fixation Solution provided with the kit for 1 h at 25 C, washed with PBS and permeabilised with the Permeabilisation Solution from the kit for 2 minutes on ice. After washing with PBS, the chips were overlaid with 100 μl of TUNEL reaction mixture, prepared according to the provider, and kept for 1 hour at 37 C in the cell incubator. After final washing with PBS, the chips were overlaid with cover slips and analysed using a fluorescence microscope.

### Microscopy and image evaluation procedure

The adenoviral spots were photographed using a microscope (Zeiss Axiovert 200 M) supported by the AxioVision LE Rel 4.5 software. The images were analyzed and evaluated using the ImageJ (Version 1.38x) software. Spot images from immunostaining with antibodies against anti-cleaved caspase 3 or after TUNEL assay were converted into 8-bit pictures and compared with shCTR images using the Image Correlator plug-in.

For the evaluation of the lamin A/C knock-down, the fluorescent signal of the markers (EGFP or RFP) was used to identify infected cells, which in turn were used to define the regions of interest (ROI). The ROI were transferred onto the images showing lamin A/C staining. This approach allows to only consider infected cells in the statistical analysis, which was performed using the StatView (SAS Institute Inc.) software.

### Caspase 3 activity assay in microwell format

The assay for caspase 3 activity was performed in a 96-well format using the Caspase-Glo 3/7™ assay (G8090, Promega) according to the manufacturer's protocol.

## Competing interests

A patent has been filed at the EPO under number EP 1 599 727 B1.

## Authors' contributions

AO performed the experimental design and data analysis. AK validated the constructs and assisted in data analysis. MT performed functional assays and drafted the manuscript. FW contributed to data acquisition and drafted the manuscript. UH performed basic viral experiments. RB, DW, and AL contributed to data acquisition, analysis, and drafting the manuscript. FW, TOJ, and HV initially designed the adenoviral microarrays. AK, MHGK, and HV suggested the study and helped interpreting the data and drafting the manuscript. HV coordinated the study and contributed most materials and resources. All authors read and approved the final manuscript.

## Supplementary Material

Additional file 1 **Storage test for the infectivity of the viruses immobilized on the chip**. **A**. Green fluorescence is indicative of successful adenoviral infection. For quantitative evaluation, the number of infected cells was counted within a 2500 μm^2 ^area of each spot. **B**. Representative images are given from microarrays stored for 1, 10 and 17 days. Scale bar, 10 μm. Click here for file
