# Supplementary figures and images for: A novel reverse transduction adenoviral array for the functional analysis of shRNA libraries
Source: BMC Genomics. 2008 Sep 24;9:441. doi: 10.1186/1471-2164-9-441 (PMC2559852; doi:10.1186/1471-2164-9-441)

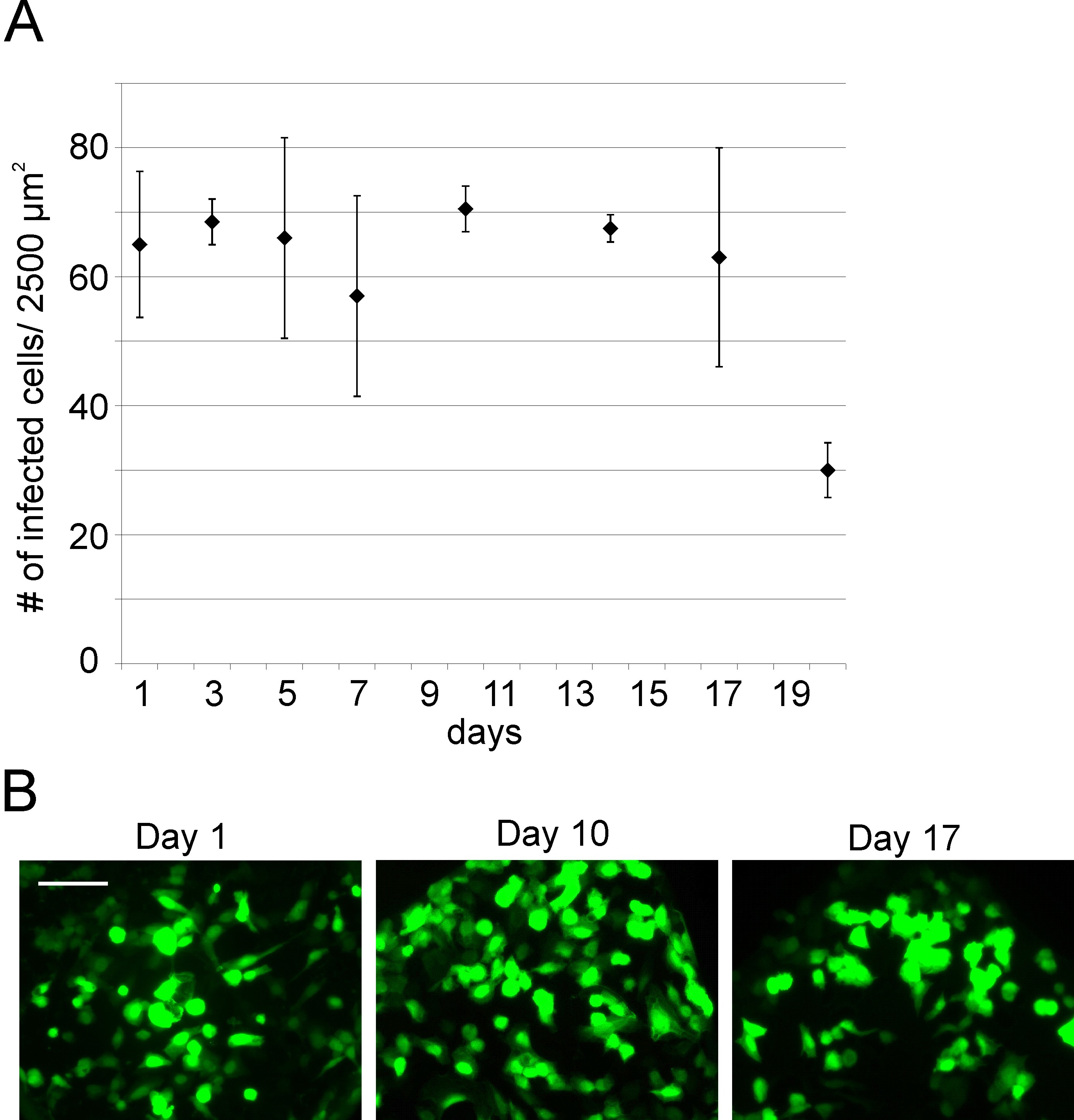

Supplement: Additional file 1 — Storage test for the infectivity of the viruses immobilized on the chip. A. Green fluorescence is indicative of successful adenoviral infection. For quantitative evaluation, the number of infected cells was counted within a 2500 μm2 area of each spot. B. Representative images are given from microarrays stored for 1, 10 and 17 days. Scale bar, 10 μm. [file 1471-2164-9-441-S1.jpeg]
